# Comparison of remimazolam and propofol combined with low dose esketamine for pediatric same-day painless bidirectional endoscopy: a randomized, controlled clinical trial

**DOI:** 10.3389/fphar.2024.1298409

**Published:** 2024-02-05

**Authors:** Tiantian Chu, Siqi Zhou, Yingfeng Wan, Qiuli Liu, Yueyang Xin, Zhang Tian, Tianqing Yan, Aijun Xu

**Affiliations:** Department of Anesthesiology, Hubei Key Laboratory of Geriatric Anesthesia and Perioperative Brain Health, and Wuhan Clinical Research Center for Geriatric Anesthesia, Tongji Hospital, Tongji Medical College, Huazhong University of Science and Technology, Wuhan, China

**Keywords:** bidirectional endoscopy, esketamine, pediatric, propofol, remimazolam, sedation

## Abstract

**Background:** Remimazolam has shown similar or even superior properties to propofol in procedural sedation in adults, but few studies have been conducted in pediatric populations. Thus, we aimed to compare the effect and safety of remimazolam and propofol combined with low dose esketamine for pediatric same-day bidirectional endoscopy (BDE).

**Methods:** Pediatrics <18 years scheduled for elective BDE under sedation were included and randomly assigned to remimazolam group (R group) or propofol group (P group). The primary outcome was the success rate of sedation. Secondary outcomes include sedation-related information and adverse events. Mean arterial pressure (MAP), heart rate (HR), and perfusion index (PI) were recorded during sedation.

**Results:** A total of 106 patients were enrolled and analyzed. The success rate of sedation was 100% in both groups. Compared with the P group, the induction time of the R group was significantly prolonged (*p* < 0.001), and the incidence of injection pain, intraoperative respiratory depression, hypotension and bradycardia was significantly lower (*p* < 0.001). The changes in MAP, HR and PI were relatively stable in the R group compared with the P group. Additionally, awake time significantly decreased with age by approximately 1.12 index points for each increase in age in the P group (*p* = 0.002) but not in the R group (*p* > 0.05). Furthermore, the decline in PI and PI ratio during BDE was related to body movement in the P group.

**Conclusion:** Remimazolam combined with low dose esketamine has a non-inferior sedative effect than propofol for pediatric BDE, with no injection pain, less respiratory depression, more stable hemodynamics. Moreover, early detection of the decline in PI may avoid harmful stimulation under light anesthesia.

**Clinical trial registration:** https://www.clinicaltrials.gov/study/NCT05686863?id=NCT05686863&rank=1, NCT05686863

## 1 Introduction

Same-day bidirectional endoscopy (BDE), including esophagogastroduodenoscopy (EGD) and colonoscopy, is commonly administered to evaluate gastrointestinal conditions (Sui et al., 2022). It has the advantages of reducing sedation times, shortening hospital stays, and reducing healthcare costs and has been increasingly applied in children, with indications including abdominal pain, vomiting, chronic diarrhoea, hematochezia, and anaemia (Urquhart et al., 2009; Hsieh et al., 2011; Isoldi et al., 2021). Given the low tolerance and compliance, pediatric gastrointestinal endoscopy tends to have a higher rate of sedation. However, there are significant distinctions between pediatric and adult endoscopy, not only in size, but also in age-related pathophysiological characteristics, including poor tolerance to hypoxia, higher sensitivity to endoscopic stimulation, smaller gastrointestinal tract, thinner gastrointestinal wall, etc., leading to a need for deep sedation in pediatric patients (Isoldi et al., 2021; Jiang, 2022).

Although there are various drugs, ideal sedation regimens for pediatric endoscopic sedation remain unclear no matter whether single sedative or combined regimen (Hayes et al., 2018; Hartjes et al., 2021). Propofol is one of the most used sedative drugs in children with the advantages of quick onset, rapid recovery, and amnesia (Zheng et al., 2022). Recently, the combination of propofol and opioids has become the preferred option for procedural sedation in many countries, but adverse events such as hypotension and respiratory depression remain a concern (Zheng et al., 2022).

Remimazolam is a novel ultra-short-acting benzodiazepine, which acts on γ-aminobutyric acid subtype A (GABA_A_) receptor and has the characteristics of water solubility, antagonism and non-irritating (Oka et al., 2021). Due to remimazolam is mainly rapidly metabolized by tissue esterases to pharmacologically inactive products, so it has the advantages of fast onset, rapid recovery, organ independence, and higher sedation quality (Kilpatrick GJ et al., 2007; Oka et al., 2021). Most clinical studies have demonstrated that remimazolam provides a high safety profile with hemodynamic stability, no respiratory depression and injection pain compared to propofol (Rex et al., 2018; Rex et al., 2021).

Given the advantages of remimazolam, it seems to be more suitable for children. However, to date, the evidence for remimazolam in pediatric gastrointestinal endoscopy is still lacking. Esketamine, an antagonist of the N-methyl-D-aspartate receptor (NMDAR), provides 2-fold higher analgesic and anesthetic potency than ketamine (Xu et al., 2022; Zhan et al., 2022). The sedative and analgesic effects of esketamine can alleviate throat irritation, and reduce consumption of sedatives and incidence of adverse events during endoscopy (Chen et al., 2022; Wang et al., 2022; Xu et al., 2022; Zhan et al., 2022; Zheng et al., 2022). Thus, we conducted a randomized, controlled trial comparing the effect and safety profiles of remimazolam and propofol combined with low dose esketamine for pediatric BDE.

## 2 Materials and methods

This is a prospective, randomized, controlled trial conducted from January to July 2023 at Tongji Hospital (Wuhan, China) and adheres to the applicable Consolidated Standards of Reporting Trials (CONSORT) guidelines. The study was approved by Tongji Medical College of Huazhong University of Science and Technology (2022S212) and registered on ClinicalTrials.gov (NCT05686863). The registration occurred prior to the start of the trial and any patient enrollment undertaken. Written informed consent was obtained from all the parents or legally authorized representatives of pediatric patients and from the patients aged 8–17 before examination.

### 2.1 Participants

Patients aged 0–17 years with American Society of Anesthesiologists (ASA) physical status I or II and scheduled for elective BDE under anesthesia were included. The exclusion criteria are as follows: patients with a high risk of full stomach and reflux aspiration, allergy to the study drug, obesity or severe malnutrition, take sedative, analgesic, or antidepressant drugs within 24 h, with untreated hypertension, with abnormal liver and kidney function, merged congenital diseases or other diseases that affect the observation of therapeutic effects, participate in other clinical studies within 4 weeks.

### 2.2 Randomization and blinding

Randomization was performed by investigators who were not involved in anesthesia management or postoperative follow-up. Patients were randomly assigned to the remimazolam group (R group) or propofol group (P group) according to the randomized number table generated by Statistical Package for Social Sciences (SPSS) software version 21.0. Randomized numbers were sealed in numbered opaque envelopes. Patients, guardians, endoscopists, and researchers responsible for intraoperative data recording and postoperative follow-up were blinded to group allocation. The anesthesiologists were the only staff who knew about the group assignments, but they were not involved in the recording and analysis of the data.

### 2.3 Intervention

All patients receive standardized care, except for experimental drugs. The intravenous access was established 30 min before the examination, and 250 mL of 5% glucose injection was infused at a rate of 8–10 mL/kg/h. 10 mL dyclonine hydrochloride mucilage was administered orally for topical anesthesia. Once attended the endoscopy room, the patient was placed in the left lateral position with the head tilted back. Blood pressure (BP), electrocardiogram (ECG), pulse oxygen saturation (SpO_2_), and perfusion index (PI) were monitored routinely. Throughout the endoscopy procedure, oxygen supplementation of 6 L/min was delivered to the nose and mouth of the patient via a threaded tube and mask.

All patients received a single dose of 0.25 mg/kg esketamine (Jiangsu Hengrui Pharmaceutical Co., Ltd., China). After 1 min, an initial bolus of 0.3 mg/kg remimazolam (Jiangsu Hengrui Pharmaceutical Co., Ltd., China) in the R group or 3 mg/kg propofol (Corden Pharma S.P.A.) in the P group were given in about 30 s at a constant speed for the induction of anesthesia. The anesthesia was maintained by continuous infusion of 1–3 mg/kg/h remimazolam or 5–10 mg/kg/h propofol and 0.5–1 μg/kg/h remifentanil (Yichang Renfu Pharmaceutical Co., Ltd., China). The BDE was performed by two experienced gastroenterologists when the Modified Observer’s Assessment of Alertness/Sedation Scale (MOAA/S) score ≤1 (Xin et al., 2022a). The endoscopy sequence was EGD followed by colonoscopy (Hsieh et al., 2011). If the patient has coughing or obvious body movement, 0.05 mg/kg remimazolam or 0.5 mg/kg propofol and 0.1 μg/kg remifentanil were added to keep quiet and painless. If the target sedation was not achieved with the sum of the initial, maintenance, and supplemental doses of remimazolam exceeding the maximum dose (12.5 mg) within a 15-min window, 0.5 mg/kg propofol was administered as a rescue sedative. The drug infusion was terminated when the colonoscopy reached the ileocecal valve in both groups. During procedural sedation, if there is apnea or SpO_2_ drop, adjust the position or lift the jaw to improve breathing. If there is significant hypotension and bradycardia, ephedrine and atropine were administered to maintain hemodynamic stability.

Once the procedure was completed, patients were transferred to the post-anesthesia care unit (PACU), where their recovery time and adverse reactions were recorded. When the modified Aldrete score was ≥9, the patient was transferred to the ward. The 24-h follow-up was conducted by a specific researcher to evaluate postoperative adverse reactions. Adverse events occurring during the examination and in the PACU were assessed and recorded by trained and blinded investigators, while those occurring in the ward within 24 h after endoscopy were reported by blinded nurses, pediatric patients and their guardians.

### 2.4 Outcomes

Demographic data and case characteristics were recorded, such as age, sex, and body mass index (BMI). The primary outcome of this study was the success rate of sedation, which was defined as a composite endpoint including completion of the procedure, no requirement for rescue sedatives, and no manual or mechanical ventilation (Borkett et al., 2015; Xin et al., 2022b).

The secondary outcomes included the procedure and sedation-relevant information, such as sedation time, induction time (from the injection of study sedatives to the disappearance of eyelash reflex), procedure time (from the insertion of gastroscope to the withdrawal of colonoscope), awake time (from the final administration of drugs to the patient’s awakening), PACU stay time. The total consumption of remimazolam, propofol, esketamine, and remifentanil was also recorded. Seven-point Likert scale was used to assess the satisfaction of endoscopists, patients and guardians ranging (from 1, or “extremely dissatisfied” to 7, or “extremely satisfied”) (Liu et al., 2021a).

Hemodynamic indicators such as mean arterial pressure (MAP), HR, and PI were recorded at the following time points: before the anesthesia induction (T0, baseline), 5 min (T1), 10 min (T2), and 15 min after induction (T3), and endoscopy examination ended (T4).

Adverse reactions during the procedure and 24-h follow-up were recorded, such as injection pain, respiratory depression (apnea or SpO_2_ < 93% over 10 s) (Zheng et al., 2022), body movement, coughing, hiccup, hypotension or hypertension (decrease or increase in MAP by 20% from baseline), bradycardia or tachycardia (decrease or increase in HR by 20% from baseline), dizziness, nausea and vomiting, visual disturbance, allergy, and abdominal distension. When hypotension or hypertension occurred, we measured again and averaged the data twice. Treatment of respiratory depression was also recorded.

### 2.5 Sample size

Based on prior research (Chen et al., 2020; Liu et al., 2021b; Chen et al., 2021; Xin et al., 2022b), the success rate of sedation ranged from 96.52% to 100% in R group and 100% in P group for procedure sedation. Therefore, we assumed 98% and 100% procedural success rates respectively in R and P groups. The predefined non-inferiority margin was set as 8%. Power Analysis and Sample Size (PASS) 15.0.5 software was used to calculate the sample size. A type I error rate of 0.05 (*α* = 0.05) and a power of 90% (*β* = 0.1) were used to estimate a sample size of 48 per group. Considering a dropout rate of 10%, we finally included 53 patients in each group.

### 2.6 Statistical analyses

SPSS software version 21.0 was used for statistical analysis. The Shapiro–Wilk test was used to identify the normality of the data distribution. Quantitative data were presented as the mean (standard deviation) (SD) or median and interquartile range (IQR). ANOVA or independent Student’s t-test was used for normally distributed data, and the Mann-Whitney U test was used for the nonparametric test. Repeated measures of ANOVA were used to test two-way interactions (group and time effects) for MAP, HR, and PI. Categorical variables were described as numbers (percentages) and analyzed by the Chi-square test or Fisher’s exact test. The odds ratio (OR) for the associations was calculated. Pearson’s and Spearman’s correlation analyses were used to detect the correlation between awake time and age, sex, BMI, sleep index, anesthesia time, or total dose of sedatives. A linear regression equation was constructed for further analysis. Point-biserial correlation coefficient (r) was applied to analyse the association between body movement and PI. *p*-value <0.05 was considered statistically significant.

## 3 Results

**FIGURE 1:**
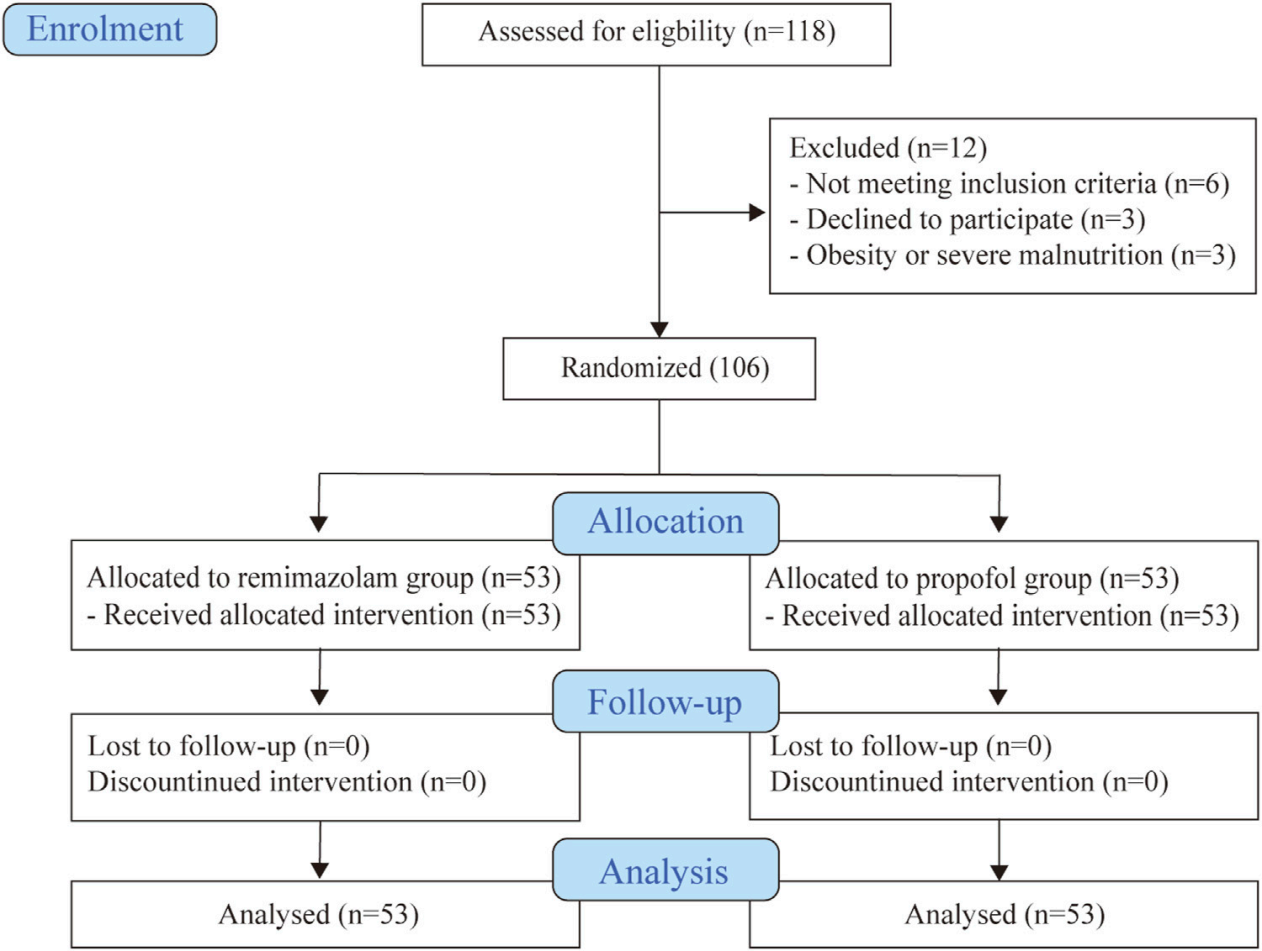
Flow diagram of included participants.

Totally 118 eligible patients were screened, and 106 subjects were enrolled and randomly assigned to either the remimazolam or propofol group (Figure 1). The baseline patient characteristics were well balanced between the two groups (Table 1). The main indication for BDE was abdominal pain in both groups (79.25% vs. 77.36%). The sleep status of the patients before the examination was similar (*p* > 0.05).

**TABLE 1:** Baseline patient characteristics.

Variable	Remimazolam group (*n* = 53)	Propofol group (*n* = 53)	*p*-value
Age (y)	10 (7–11)	10 (7–12)	0.461^a^
Sex			0.319^b^
Male, n (%)	30 (56.60)	35 (66.04)	
Female, n (%)	23 (43.40)	18 (33.96)	
Weight (kg)	31.5 (26.6–43)	30.0 (25–43.5)	0.582^a^
Height (cm)	140.08 ± 17.04	139.26 ± 19.39	0.819^c^
BMI (kg/m^2^)	16.46 (15.19–19.02)	15.68 (14.38–17.35)	0.274^a^
ASA physical status			>0.999^b^
I, n (%)	43 (81.13)	43 (81.13)	
II, n (%)	10 (18.87)	10 (18.87)	
Indication			0.795^d^
Abdominal pain, n (%)	42 (79.25)	41 (77.36)	
Vomit, n (%)	5 (9.43)	7 (13.21)	
Hematochezia, n (%)	2 (3.77)	3 (5.66)	
Other, n (%)	4 (7.55)	2 (3.77)	
Sleep time before endoscopy (h)	6.00 ± 1.51	6.28 ± 1.60	0.368^c^
Sleep Index	0.70 ± 0.19	0.74 ± 0.17	0.225^c^

*BMI*, body mass index; *ASA*, american society of anesthesiologists.

Data were expressed by mean ± SD, median (IQR), or frequencies and percentages.

^a^ Mann–Whitney U test.

^b^ Chi-square test.

^c^ *T*-test.

^d^ Corrected Chi-square test.

### 3.1 Primary outcome and sedation-related outcomes

The success rate of sedation during the pediatric BDE was 100% between the R and P groups. The difference in rate (R vs. P) was 0, with the lower confidence limit not crossing the non-inferiority limit of 8%. There were no significant differences in sedation time, procedure time and PACU stay time between the two groups (*p* > 0.05) (Table 2). However, the induction time in the R group was significantly longer than that of the P group (*p* < 0.001). During the whole procedure, although the consumption of remifentanil (4.46 [3.99-5.47]) was higher in the R group compared with the P group (4.23 [3.52-5.04]), there were no significant differences between the two groups (*p* > 0.05). In addition, the endoscopists, patients, and guardians had similar sedation satisfaction scores with the two groups.

**TABLE 2:** Procedure and sedation-related conditions.

Variable	Remimazolam group (*n* = 53)	Propofol group (*n* = 53)	*p*-value
Sedation time (min)	21.93 ± 5.72	23.40 ± 5.62	0.184^a^
Induction time (sec)	52 (45–61)	30 (24–36)	<0.001^b^
Procedure time (min)	19.30 ± 5.58	20.87 ± 5.64	0.154^a^
Awake time (min)	21.91 ± 10.67	22.53 ± 8.03	0.735^a^
PACU stay time (min)	23.42 ± 11.86	24.36 ± 10.74	0.669^a^
Administered dose of anesthetics			
Remimazolam (mg)	19.09 ± 6.18		
Propofol (mg)		191.52 ± 74.48	
Esketamine (mg)	7.81 (6.31–10.75)	7.50 (6.25–10.75)	0.584^b^
Remifentanil (μg)	45 (38.48–57.20)	47.20 (35.05–58.85)	0.786^b^
Remifentanil (μg • kg^-1^ • h^-1^)	4.46 (3.99–5.47)	4.23 (3.52–5.04)	0.191^b^
Satisfaction for sedation			
Endoscopist	7 (7–7)	7 (7–7)	0.959^b^
Patients	7 (7–7)	7 (7–7)	0.634^b^
Guardians	7 (6–7)	7 (6–7)	0.927^b^

*PACU*, postanesthesia care unit.

^*a*^ *T-test.*

^b^ Mann–Whitney U test.

**FIGURE 2:**
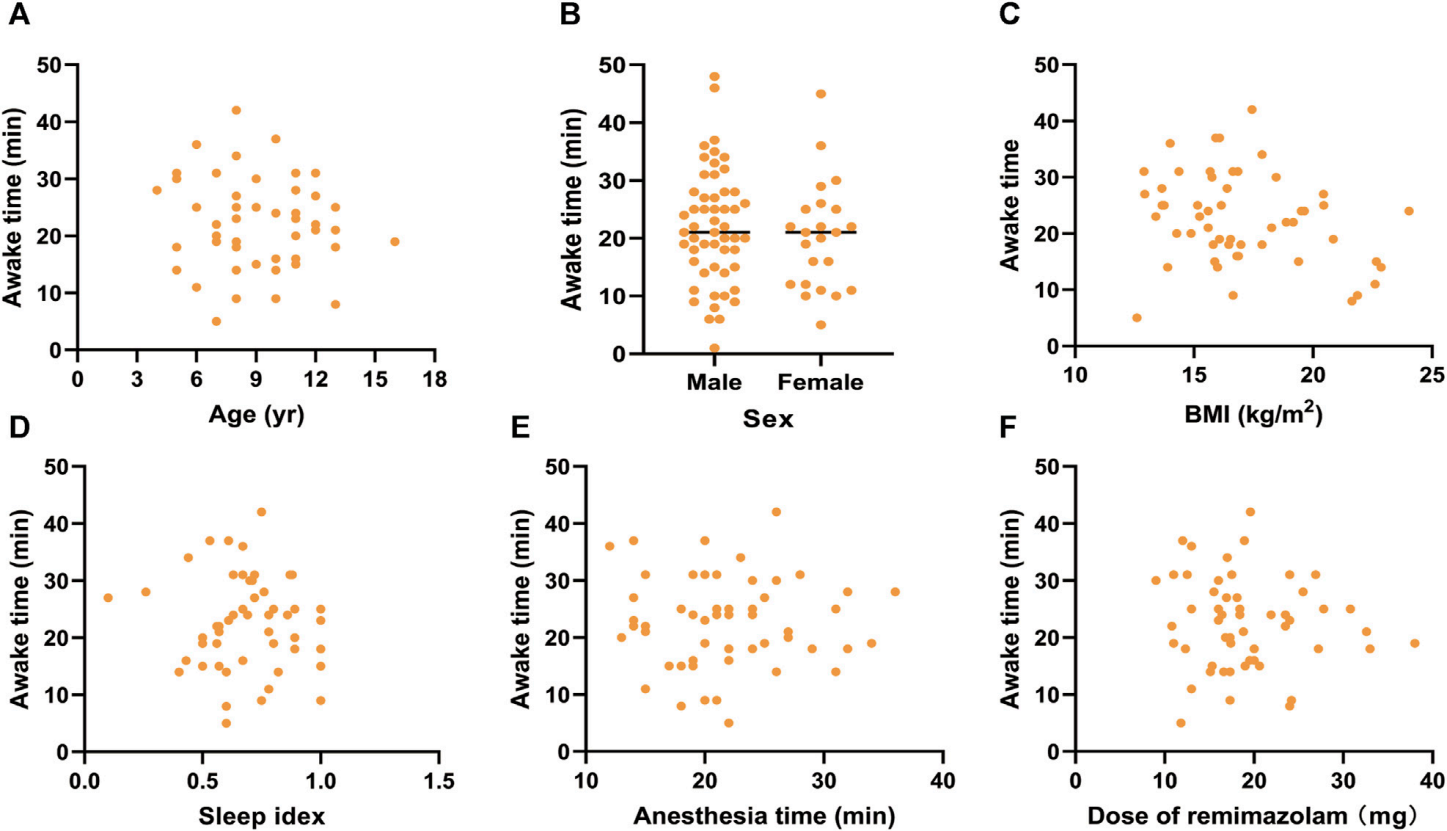
Scatter plots for awake time and **(A)** age, **(B)** sex, **(C)** BMI, **(D)** sleep index, **(E)** anethesia time, and **(F)** Dose of remimazolam under remimazolam anesthesia. BMI, body mass index.

**FIGURE 3:**
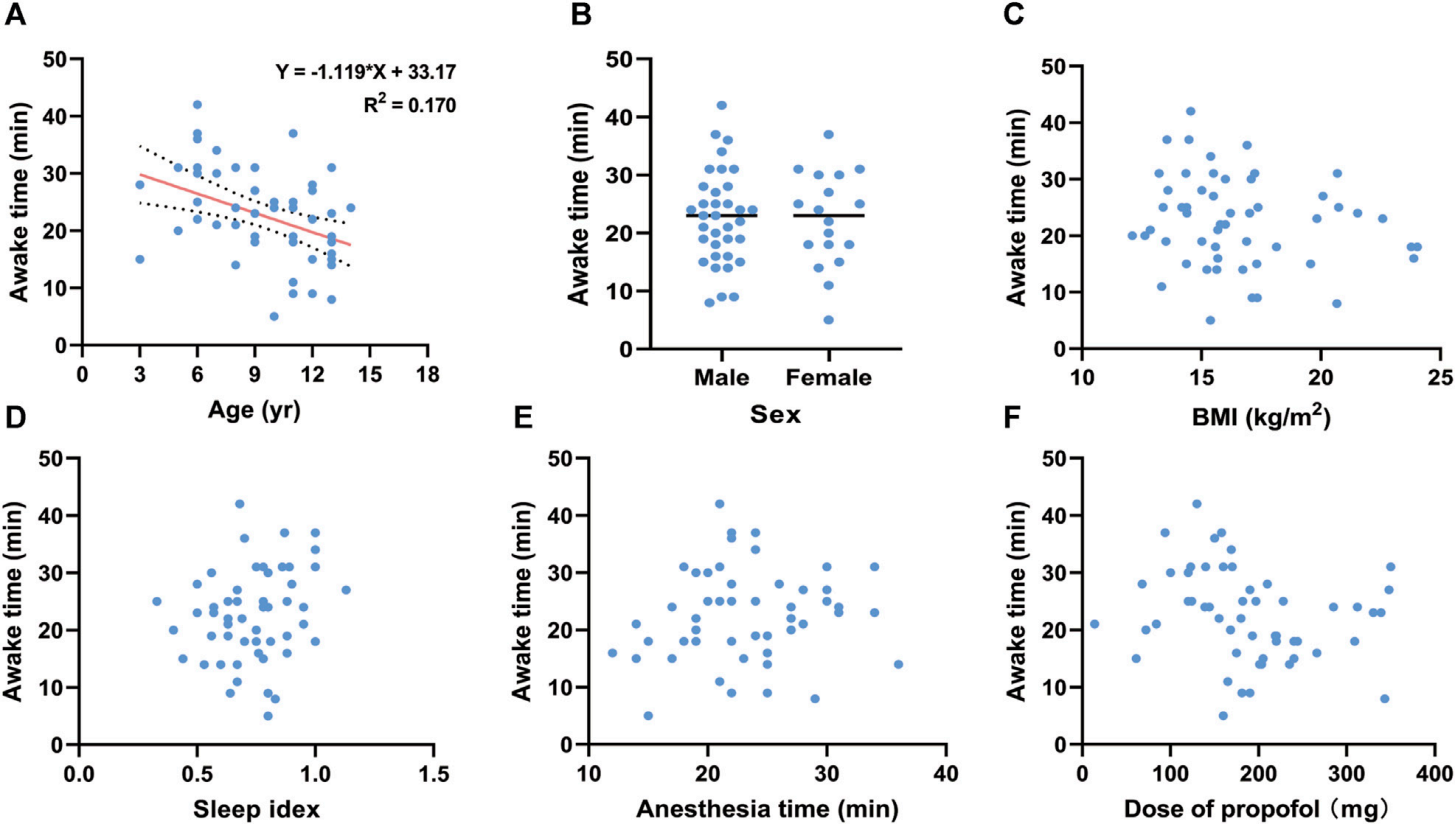
Linear regression for awake time and **(A)** age under propofol anesthesia. Scatter plots for awake time and **(B)** sex, **(C)** BMI, **(D)** sleep index, **(E)** anethesia time, and **(F)** Dose of propofol under propofol anesthesia. BMI, body mass index.

To investigate the influencing factors of awake time, we conducted linear correlation analyses and generated scatter plots (Figures 2, 3). There were no correlations between awake time and age (*r* = −0.118, *p =* 0.399), sex (*r* = −0.133, *p =* 0.341), BMI (*r* = −0.054, *p =* 0.700), sleep index (*r* = −0.046, *p =* 0.742), anesthesia time (*r* = 0.064, *p =* 0.647), or total dose of remimazolam (*r* = −0.199, *p =* 0.152) in the R group. However, in the P group, a negative correlation between awake time and age (*r* = −4.27, *p* = 0.001) was observed. A linear regression equation was constructed (*F* = 10.453, *p* = 0.002) and the results demonstrated that awake time significantly decreased with age by approximately 1.12 index points for each increase in age under propofol anesthesia [β =—1.119, 95% CI (−1.813, −0.424); *p* = 0.002].

### 3.2 Adverse events

Adverse events were summarized in Table 3. During anesthesia induction, 14 patients (26.42%) in the P group had painful injections (*p* < 0.001). The incidence of respiratory depression in R group (9.43%) was significantly lower than that in P group (35.85%) (OR, 0.186; 95% CI, 0.063 to 0.548; *p* = 0.001), and most respiratory depression can be relieved by adjusting the position and lifting the jaw in both groups (100% vs. 89.47%). During the procedure, the incidence of hypotension (18.89% vs. 45.28%) (OR, 0.281; 95% CI, 0.117 to 0.674; *p* = 0.006) and bradycardia (1.89% vs. 30.19%) (OR, 0.044; 95% CI, 0.006 to 0.350; *p* < 0.001) in R group was lower than that in P group. Although body movement, coughing/hiccup, and tachycardia were more likely to be observed in the R group, there was no statistical difference between the two groups (*p* > 0.05). Subgroup analysis was performed for respiratory depression, hypotension, and bradycardia according to age, but the results should be treated with caution due to the small sample size of each subgroup (Supplementary Table S1). Dizziness is the most common adverse event in PACU, occurring in 9 patients (16.98%) of R group and 5 patients (9.43%) of P group (*p* = 0.390). In addition, bradycardia, nausea and vomiting, and visual impairment were also observed in PACU (all *p* > 0.999). After 24 h of the procedure, 5 patients (9.43%) in the R group reported dizziness (*p* = 0.067) and 2 patients (3.77%) had fever (<38°C) (*p* > 0.999), while 1 patient (1.89%) in the P group reported allergy (*p* > 0.999), manifested as red rash and pruritus, and 1 patient (1.89%) had fever (<38°C). In addition, there were no serious adverse events requiring flumazenil antagonism during the study.

**TABLE 3:** Analysis of adverse events.

Variable	Remimazolam group (*n* = 53)	Propofol group (*n* = 53)	*p*-value
**After induction**			
Painful injection, n (%)	0	14 (26.42)	<0.001^a^
Respiratory depression, n (%)	5 (9.43)	19 (35.85)	0.001^a^
Adjusting posture, n (%)	4 (7.55)	12 (22.64)	0.055^a^
Jaw lift, n (%)	1 (1.89)	5 (9.43)	0.205^b^
Bag-Mask Ventilation, n (%)	0	2 (3.77)	0.495^c^
Endotracheal intubation, n (%)	0	0	>0.999^c^
**Intra-examination**			
Body movement, n (%)	10 (19.87)	8 (15.09)	0.797^a^
Coughing/Hiccup, n (%)	5 (9.43)	1 (1.89)	0.207^b^
Hypotension, n (%)	10 (18.89)	24 (45.28)	0.006^a^
Tachycardia, n (%)	8 (15.09)	2 (3.77)	0.093^a^
Bradycardia, n (%)	1 (1.89)	16 (30.19)	<0.001^a^
**In the PACU**			
Bradycardia, n (%)	0	1 (1.89)	>0.999^c^
Nausea and vomiting, n (%)	2 (3.77)	2 (3.77)	>0.999^b^
Dizziness, n (%)	9 (16.98)	5 (9.43)	0.390^a^
Visual disturbance, n (%)	2 (3.77)	1 (1.89)	>0.999^b^
**24 h after procedure**			
Dizziness, n (%)	5 (9.43)	0	0.067^b^
Fever, n (%)	2 (3.77)	1 (1.89)	>0.999^b^
Allergy, n (%)	0	1 (1.89)	>0.999^c^
Abdominal distension, n (%)	3 (5.7)	3 (5.7)	>0.999^b^
Nausea and vomiting, n (%)	0	0	>0.999^c^

*PACU*, postanesthesia care unit.

^a^ Chi-square test.

^b^ Corrected Chi-square test.

^c^ Fisher’s exact test.

### 3.3 Hemodynamic results

**FIGURE 4:**
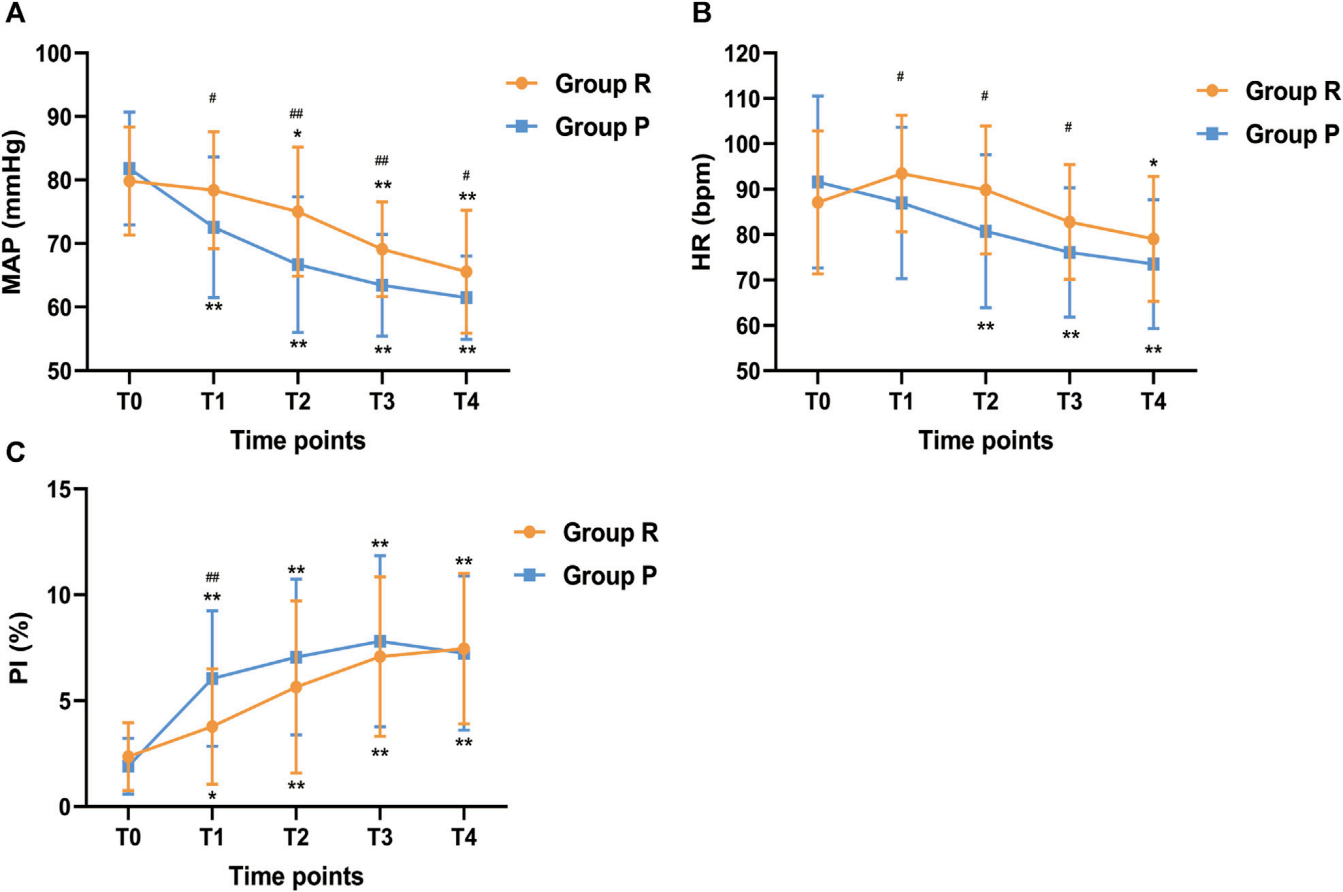
Changes in **(A)** MAP, **(B)** HR, and **(C)** PI across different study time points. MAP, Mean arterial pressure; HR, Heart rate; PI, perfusion index. Before anesthesia induction (T0; baseline); 5 min after induction (T1); 10 min after induction (T2); 15 min after induction (T3); endoscopy examination ended (T4). ^*^ *p* < 0.05 and ^**^ *p* < 0.001 compared with baseline (T0), ^#^ *p* < 0.05 and ^##^ *p* < 0.001 compared with P group.

The changes in vital signs were shown in Figure 4. Compared with T0, the MAP of the R group had no significant change at T1 (*p* = 0.988) but continued to decline at T2 (*p* = 0.017), T3 (*p* < 0.001) and T4 (*p* < 0.001), while MAP in the P group began to decrease from T1 and was lower than that in the R group (*p* < 0.05). At T1, T2 and T3, the HR of the R group were significantly higher than that of the P group, while compared with baseline, there was no significant difference. After anesthesia induction, PI was increased both in the R group and P group compared with baseline (*p* < 0.05), but at T1, PI in the P group was significantly higher than that in the R group (*p* < 0.001). Hence, the changes in MAP, HR and PI showed that the hemodynamic fluctuation was relatively stable in the R group.

### 3.4 Body movements and PI

A total of 14 body movements occurred in the R group (Table 4). Although PI and PI ratio within 5 min before body movement were higher than those during body movement, there were no significant differences (all *p* > 0.05). Besides, Point-biserial correlation analysis showed that body movement was not correlated with PI (*r* =−0.348, *p* = 0.070), but with PI ratio (*r* =−0.375, *p* = 0.049). In contrast, 12 body movements occurred in the P group. PI and PI ratio were significantly higher before body movement than during body movements (*p* = 0.001; *p* = 0.002). Furthermore, correlation analysis also showed that there were negative correlations between body movement and PI (r =−0.689, *p* < 0.001) and PI ratio (r =−0.622, *p* = 0.001).

**TABLE 4:** The correlation between body movement and PI and PI ratio.

	Remimazolam group(*n* = 14)			Propofol group(*n* = 12)		
	Before body movement	Body movement	*p*-value	Before body movement	Body movement	*p*-value
PI	3.11 ± 3.53	1.28 ± 0.84	0.070	4.63 ± 2.45	1.38 ± 0.58	0.001
PI ratio	2.04 ± 2.27	0.76 ± 0.48	0.058	3.01 ± 1.93	0.98 ± 0.61	0.002

*PI*, perfusion index.

## 4 Discussion

This study aimed to compare the efficacy and safety of remimazolam and propofol combined with low dose esketamine for pediatric BDE. The results demonstrated that the success rate of sedation in both groups was 100%. The induction time in the remimazolam group was prolonged, but the incidence of injection pain and respiratory depression was reduced, and patients showed higher hemodynamic stability during the examination. Additionally, it was observed that the awake time in the propofol group demonstrated a negative correlation with age, whereas individual characteristics were not found to have a significant impact on awake time in the remimazolam group.

Propofol is the most commonly used sedative for pediatric sedation, but side effects limit its use. Remimazolam has demonstrated similar or even superior properties to propofol in procedural sedation in adults for its safety and effectiveness (Sheng et al., 2020; Zhu et al., 2021; Xin et al., 2022a; Guo et al., 2022). In our previous study, we compared remimazolam and propofol for colonoscopic polypectomy in adults. The results showed that remimazolam has a non-inferior sedative effect than propofol and might be a safer alternative (Xin et al., 2022b). In addition, due to the characteristics of rapid conversion to inactive metabolites, remimazolam may reduce cumulative neurotoxicity, which will benefit pediatric patients compared to other commonly used anesthetics (Useinovic and Jevtovic-Todorovic, 2022).

Regarding the dose selection of the drugs, the initial dose range of remimazolam for procedural sedation or anesthesia in adults in previous studies was 0.1–0.6 mg kg^-1^ (Zhang et al., 2022). In our pilot study, we evaluated initial doses of 0.1, 0.2, and 0.3 mg kg^-1^ remimazolam to induce anesthesia, and observed that 0.1 and 0.2 mg kg^-1^ were insufficient for sedation, while 0.3 mg kg^-1^ remimazolam achieved effective sedation without significant adverse effects. Based on these findings, we determined the induction dose of remimazolam to be 0.3 mg kg^-1^, with a maintenance dose of 1–3 mg kg^-1^•h^-1^. This dosing regimen aligns with both the medication’s instruction and the regimen used in a multicenter study of general anesthesia in pediatric patients (Fang et al., 2023). Additionally, the administered dose of propofol was selected based on the standard dosing in our centre, aiming to effectively inhibit most stress responses to procedural stimuli and achieve effective sedation.

A meta-analysis showed that remimazolam had a lower success rate of sedation/general anesthesia than propofol (Zhang et al., 2022). However, in our study, remimazolam was considered non-inferior to propofol in sedative efficacy, probably due to the different doses we used and the combination of esketamine. In addition, we found that the induction time in R group was longer than that in P group, but there was no difference in awake time and PACU stay time. Similar results were also obtained in previous studies (Chen et al., 2020; Tang et al., 2022; Zhang et al., 2022). However, there are also some studies with inconsistent results. For example, Dong et al. compared remimazolam to propofol for endoscopic retrograde cholangiopancreatography and found that the induction time of remimazolam sedation was no different from that of propofol, while the awake time and recovery time were prolonged (Dong et al., 2023). These opposing results may be attributed to variations in the types of procedures, doses of anesthetics, and the populations included in the studies (Canpolat et al., 2016). Furthermore, we observed that awake time in the R group was not influenced by factors such as age, sex, BMI, sleep index, anesthesia time, and total dose of remimazolam. This finding aligns with the known pharmacological characteristics of remimazolam, which is less dependent on individual patient characteristics and has no cumulative sedative effects (Kilpatrick, 2021; Useinovic and Jevtovic-Todorovic, 2022).

Respiratory depression is one of the serious side effects of propofol sedation, often leading to hypoxemia, especially in children who are more sensitive to hypoxia. Most studies have shown that compared with commonly used propofol sedation, remimazolam reduces the risk of respiratory depression during endoscopic procedures (Zhu et al., 2021; Tang et al., 2022; Zhang et al., 2022). Similarly, in this study, the incidence of respiratory depression was significantly reduced in the R group, most of which was relieved by position adjustment or jaw thrust. This indicates remimazolam also has a milder respiratory depression than propofol in children.

Due to the cardiovascular inhibitory effect of propofol, it often causes adverse reactions such as hypotension and bradycardia (Xin Y. et al., 2022). However, to date, clear criteria for hypotension in children do not exist. A study showed that the blood pressure of children decreased significantly after anesthesia (de Graaff et al., 2016). The lower limits (-2SD) of the reference values of MAP were 17 mmHg at birth and 47 mmHg at 18 years old. In our study, we defined hypotension as MAP less than 20% of the baseline value based on previous studies (Erden et al., 2009; Zheng et al., 2022), and 45.28% of the patients had intraoperative hypotension in P group. The high incidence may be related to the definition of hypotension but was significantly higher than in R group. Similarly, the incidence of bradycardia in P group was higher than in R group. These results suggest that remimazolam has a weaker cardiovascular inhibitory effect in pediatrics than propofol and can avoid sharp fluctuations in hemodynamics. The changes of MAP, HR, and PI more intuitively prove this conclusion.

PI is the ratio of pulsatile and non-pulsatile blood flow, which reflects changes in vascular resistance and is mainly affected by sympathetic tone (Chu et al., 2023). PI ratio is calculated as the ratio of PI at each moment to baseline PI, which can reduce individual variability. Studies have shown that PI can be well used to monitor the depth of anesthesia and predict awakening (Skowno, 2013; Enekvist and Johansson, 2015; Krishnamohan et al., 2016; Liu et al., 2018). Otherwise, PI is also considered a sensitive indicator reflecting sympathetic nerve activity to pain stimulation (Enekvist and Johansson, 2015). Body movement is often caused by insufficient sedation or analgesia resulting in involuntary limb movement, following increased sympathetic nerve excitability (Ezri T et al., 1998; Kupeli and Kulhan, 2018). Therefore, the rapid change of PI may have the opportunity to be a predictor of body movement. In this study, the PI and PI ratio of patients in the P group decreased significantly when body movement occurred, while that in the R group showed a downward trend, but the difference was not statistically significant, which may be related to the small sample size and the strong and rapid effect of propofol on cardiovascular inhibition. PI and PI ratio dropped near to preanesthetic levels probably associated with sympathetic excitation and increased vascular tone under light anesthesia. Besides, correlation analysis suggested that body movement and PI in P group, and body movement and PI ratio in both groups were negatively correlated. Hence, this study suggests that the dramatic decline in PI and PI ratio during BDE was related to body movement, and early detection of the decline in PI may avoid the harmful stimulation under light anesthesia, but this finding needs to be further verified by larger sample size studies.

Studies have shown that esketamine combined with propofol or remimazolam for procedural sedation can maintain stable hemodynamics and reduce the incidence of adverse events (Chen et al., 2022; Xu et al., 2022; Yi et al., 2022; Zhan et al., 2022; Zheng et al., 2022). Zhao et al. reported that 84.8% of injection pain occurred in pediatric patients with propofol, which could be reduced by ketamine pretreatment (Zhao et al., 2012). In this study, the incidence of injection pain in P group was 26.42% when combined with low dose esketamine, which indicates a low dose of esketamine has similar effects to ketamine and can effectively prevent pain after propofol injection. However, the awake time in this study appeared to be longer than in studies with propofol or remimazolam sedated alone (Zhang et al., 2021; Xin et al., 2022b), while similar to studies in combination with esketamine (Wang et al., 2022; Zheng et al., 2022). Dizziness and visual disturbance were also observed in both groups and could be attributed to esketamine (Li et al., 2022; Wang et al., 2022; Zhan et al., 2022), but they were self-limited and did not require intervention.

There are still some limitations in this study. First, this study was conducted in a single centre, so larger studies are still needed to verify the conclusions. Second, we included only children with ASA I/Ⅱ grade, so the safety of remimazolam in critically ill children should be evaluated in additional studies. Third, we did not use bispectral index (BIS) to monitor the depth of anesthesia during endoscopic procedures. Although BIS is the most commonly used anesthesia depth monitoring technique, its reliability in the pediatric population is still controversial (Tirel et al., 2008; Kim et al., 2022; Liu et al., 2022).

## 5 Conclusion

In conclusion, remimazolam combined with low dose esketamine is non-inferior to propofol in terms of sedative efficacy for pediatric BDE, with no injection pain, less respiratory depression, and more stable hemodynamics. Awake time was negatively associated with age in the propofol group but not in the remazolam group. In addition, the rapid decrease in PI seems to correlate well with body movement, and future studies could explore whether PI can be used as a predictor of body movement during procedural sedation.

## Data Availability

The original contributions presented in the study are included in the article/Supplementary Material, further inquiries can be directed to the corresponding author.

## Abbreviations

ASA, American Society of Anesthesiologists; BDE, bidirectional endoscopy; BIS, bispectral index; BMI, body mass index; BP, blood pressure; CI, confidence interval; CONSORT, Consolidated Standards of Reporting Trials; ECG, electrocardiogram; EGD, esophagogastroduodenoscopy; GABA_A_, γ-aminobutyric acid subtype A; IQR, interquartile range; MAP, mean arterial pressure; MOAA/S, Modified Observer’s Assessment of Alertness/Sedation Scale; OR, odds ratio; PACU, post-anesthesia care unit; PASS, Power Analysis and Sample Size; PI, perfusion index; SD, standard deviation; S_P_O_2_, pulse oxygen saturation; SPSS, Statistical Package for Social Sciences.
