# Supplementary material for: Comparison of remimazolam and propofol combined with low dose esketamine for pediatric same-day painless bidirectional endoscopy: a randomized, controlled clinical trial
Source: Front Pharmacol. 2024 Feb 5;15:1298409. doi: 10.3389/fphar.2024.1298409 (PMC10875078; doi:10.3389/fphar.2024.1298409)
Supplement: Supplementary file 1 [file Table1.DOCX]

**Supplementary material**

**Supplementary table 1.** Subgroup analyses of main adverse events according to ages.

|  | Remimazolam group | Propofol  group | | OR (95% CI) | *P*-value |
| --- | --- | --- | --- | --- | --- |
| Respiratory depression | | | | | |
| Age (yr) | | | | | |
| 0-6 | 1/9 | | 4/12 | 0.25 (0.02-2.76) | 0.506 |
| 7-12 | 3/38 | | 12/30 | 0.13 (0.03-0.52) | 0.003 |
| 13-18 | 1/6 | | 3/11 | 0.53 (0.04-6.66) | 0.615 |
| Hypotension | | | | | |
| Age (yr) |  | |  |  |  |
| 0-6 | 0/9 | | 6/12 | 2.00 (1.14-3.52) | 0.043 |
| 7-12 | 8/38 | | 14/30 | 0.31 (0.11-0.88) | 0.037 |
| 13-18 | 2/6 | | 4/11 | 0.88 (0.11-7.11) | >0.999 |
| Bradycardia | | | | | |
| Age (yr) | | | | | |
| 0-6 | 0/9 | | 4/12 | / | 0.173 |
| 7-12 | 0/38 | | 8/30 | / | 0.03 |
| 13-18 | 1/6 | | 4/11 | 0.35 (0.03-4.15) | 0.768 |

Data are presented as positives/totals.
